# Magnetic resonance imaging and genetic investigation of a case of rottweiler leukoencephalomyelopathy

**DOI:** 10.1186/1746-6148-9-57

**Published:** 2013-03-26

**Authors:** Katrin Hirschvogel, Kaspar Matiasek, Katharina Flatz, Michaela Drögemüller, Cord Drögemüller, Bärbel Reiner, Andrea Fischer

**Affiliations:** 1 Department of Veterinary Clinical Sciences Ludwig-Maximilians-Universitaet, Neurology Service, Clinic of Small Animal Medicine, Munich, Germany; 2 Department of Veterinary Clinical Sciences Ludwig-Maximilians-Universitaet, Section of Clinical & Comparative Neuropathology, Institute of Veterinary Pathology, Munich, Germany; 3 Department of Veterinary Clinical Sciences Ludwig-Maximilians-Universitaet, Clinic of Small Animal Surgery and Reproduction, Munich, Germany; Small Animal Hospital Hüttig, Reutlingen, Germany; 4 Institute of Genetics, Vetsuisse Faculty, University of Berne, Berne, Switzerland

**Keywords:** Rottweiler, *DARS2*, LBSL, White matter disease, Progressive ataxia

## Abstract

**Background:**

Leukoencephalomyelopathy is an inherited neurodegenerative disorder that affects the white matter of the spinal cord and brain and is known to occur in the Rottweiler breed. Due to the lack of a genetic test for this disorder, post mortem neuropathological examinations are required to confirm the diagnosis. Leukoencephalopathy with brain stem and spinal cord involvement and elevated lactate levels is a rare, autosomal recessive disorder in humans that was recently described to have clinical features and magnetic resonance imaging (MRI) findings that are similar to the histopathologic lesions that define leukoencephalomyelopathy in Rottweilers. Leukoencephalopathy with brain stem and spinal cord involvement is caused by mutations in the *DARS2* gene, which encodes a mitochondrial aspartyl-tRNA synthetase. The objective of this case report is to present the results of MRI and candidate gene analysis of a case of Rottweiler leukoencephalomyelopathy to investigate the hypothesis that leukoencephalomyelopathy in Rottweilers could serve as an animal model of human leukoencephalopathy with brain stem and spinal cord involvement.

**Case presentation:**

A two-and-a-half-year-old male purebred Rottweiler was evaluated for generalised progressive ataxia with hypermetria that was most evident in the thoracic limbs. MRI (T2-weighted) demonstrated well-circumscribed hyperintense signals within both lateral funiculi that extended from the level of the first to the sixth cervical vertebral body. A neurodegenerative disorder was suspected based on the progressive clinical course and MRI findings, and Rottweiler leukoencephalomyelopathy was subsequently confirmed via histopathology. The *DARS2* gene was investigated as a causative candidate, but a sequence analysis failed to identify any disease-associated variants in the DNA sequence.

**Conclusion:**

It was concluded that MRI may aid in the pre-mortem diagnosis of suspected cases of leukoencephalomyelopathy. Genes other than *DARS2* may be involved in Rottweiler leukoencephalomyelopathy and may also be relevant in human leukoencephalopathy with brain stem and spinal cord involvement.

## Background

Rottweiler leukoencephalomyelopathy (LEM) was initially recognised in the US as a cause of chronic progressive ataxia with insidious onset in Rottweilers between 1.5 and 4 years of age [1]. The clinical and pathological characteristics of this disease entity were further defined in subsequent reports originating from Australia, the Netherlands and the UK, which described 16 pathologically confirmed cases (of 22 total cases described in the literature) and suggested an autosomal recessive pattern of inheritance [2-6]. In these reports, Rottweiler LEM presented as a distinctive neurodegenerative disorder restricted to the lateral and dorsal funiculi of the cervical spinal cord and spinal tracts of the trigeminal nerve, pyramids, caudal cerebellar peduncles, cerebellar medulla and optic tracts that showed a sharp demarcation between abnormal and normal white matter and occasional microcavitation in the centre of the lesion. Clinically, affected dogs exhibit progressive ataxia with hypermetria and subtle postural reaction deficits. Thus far, the ante mortem diagnosis of LEM in Rottweilers has been based on clinical suspicion and the exclusion of other diseases of the cervical spinal cord, e.g., compression/instability, neoplasia and inflammation. To date, there have been no magnetic resonance imaging (MRI) studies or genetic investigations of this disease entity.

Leukoencephalopathy with brain stem and spinal cord involvement and lactate elevation (LBSL) is a neurodegenerative disease in humans with clinical features and MRI findings that are surprisingly similar to the histopathologic lesions of LEM in Rottweilers. Specifically, these patients exhibit slow progressive spasticity and ataxia, MRI findings of selective involvement of the brain stem and spinal tracts in both lateral funiculi and dorsal columns and changes in the cerebral and cerebellar white matter. Spinal cord involvement with MR signal intensity changes has also been reported in other leukodystrophies in humans, e.g., adult onset autosomal dominant leukodystrophy with autonomic features, Alexander’s disease, vitamin B12 deficiency myelopathy and sporadic cases of adult onset lysosomal leukodystrophies [7-11]; however, a very distinct and well-demarcated pattern of signal intensity change is considered to be most characteristic of LBSL. In LBSL, high levels of lactate are frequently demonstrated in brain lesions using magnetic resonance (MR) spectroscopy; this finding suggests a respiratory chain defect, but lactate is rarely elevated in the blood or cerebrospinal fluid (CSF) [12,13]. To date, all human cases of LBSL have been found to be caused by mutations in the *DARS2* gene, which encodes mitochondrial aspartyl-tRNA synthetase [14,15].

To investigate the hypothesis that LEM in Rottweilers could represent a possible animal model of LBSL, MRI results and *DARS2* gene integrity were investigated in a single, affected dog.

## Case presentation

A two-and-a-half-year-old male purebred Rottweiler was referred for further investigation of progressive ataxia. The dog had been placed in an animal shelter 8 weeks prior to the study. Unfortunately, no pedigree data were available, and we were unable to ascertain whether inbreeding had occurred. At the time of shelter placement, the dog had already been ataxic, and the ataxia progressed during the subsequent 8 weeks. Haematologic and serum biochemical analyses, thoracic and abdominal radiographs, and echocardiography had been performed prior to referral, and the findings were unremarkable.

Physical examination showed excessive wearing of the nails on all four limbs, particularly of the thoracic limbs. A neurological examination showed severe generalised ataxia with hypermetria of the thoracic (prolonged protraction and overreaching action with limb extension) and pelvic limbs. Additionally, difficulties in rising, intermittent crossing of the thoracic limbs, and a wide-based stance of all limbs were observed. The postural reactions (wheelbarrowing with and without neck extension, hopping, and proprioceptive positioning) were delayed, and the thoracic limbs were more severely affected than the pelvic limbs. A supplemental movie file shows these findings in more detail (see Additional file 1). The spinal reflexes (extensor carpi radialis, thoracic and pelvic limb flexor, patellar, cranial tibial, gastrocnemius, cutaneous trunci, and perineal) were all normal. The mentation and cranial nerve function, including vision, were unimpaired, but an inconsistent menace response was observed; this was attributed to the lack of cooperation by the dog but could also indicate a cerebellar lesion. Palpation of the head and spine and neck flexion and extension did not elicit any signs of pain. There was no evidence of tremor or uncoordinated movements of the head. The findings of the neurological examination were most consistent with a cervical myelopathy (C1-C5 spinal cord segments) involving the spinocerebellar tracts, although a cerebellar lesion could not be ruled out completely. The differential diagnoses included several breed-related neurodegenerative disorders: neuronal vacuolation and spinocerebellar degeneration, neuroaxonal dystrophy, LEM, cervical spondylomyelopathy and arachnoid diverticula [16-20].

**Figure 1:**
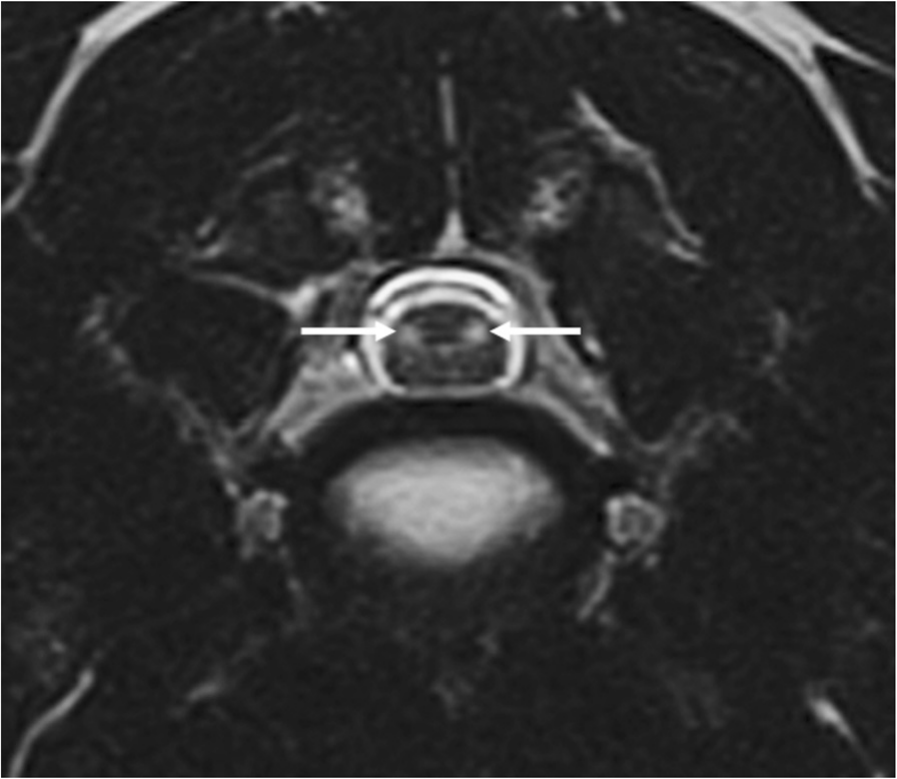
**Transverse T2-weighted MR images of the cervical spinal cord at the level of the C4-C5 intervertebral disc space.** The images show well-demarcated, ovoid, hyperintense signals with a bilateral, symmetrical appearance in the region of the lateral funiculi (arrow).

**Figure 2:**
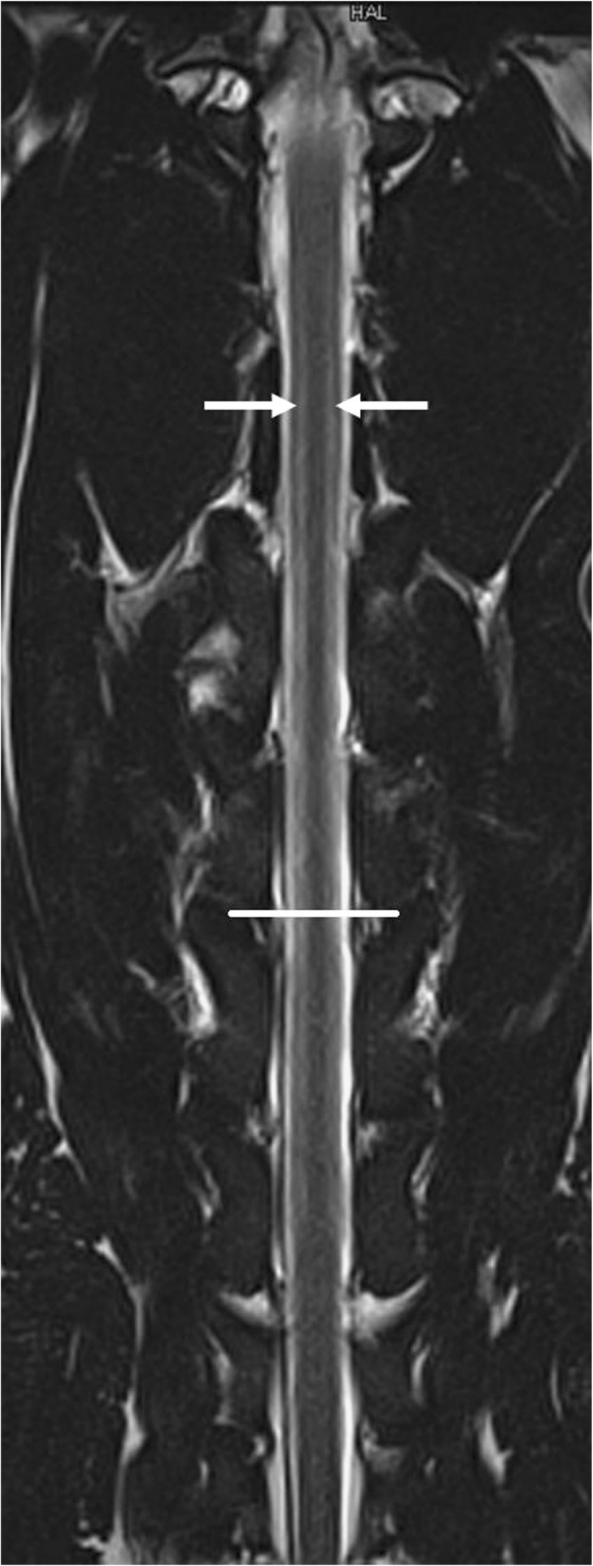
**Dorsal T2-weighted MR images of the cervical spinal cord.** The image shows linear, hyperintense signals (arrow) corresponding to the lesions in Figure 1 that extend from the level of the first to the sixth cervical vertebral body in a bilateral, symmetrical fashion. The line denotes the C4-C5 intervertebral disk space.

A follow-up laboratory examination revealed mild eosinophilia (1.51 × 10^3^ eosinophilic granulocytes/μl; reference range: 0.04 - 0.6 × 10^3^/μl) and unremarkable serum biochemical results. The dog was subsequently anesthetised for further examination of the cervical spine and brain using MRI and CSF analysis. Electrodiagnostic examination was scheduled as a supplemental examination to investigate the presence of additional lesions in the peripheral nerves. Magnetic resonance imaging was performed using a 1.5 T magnetic resonance unit. The brain imaging protocol utilised sagittal, dorsal and transverse T2-weighted (TR/TE 5190/108 ms) and T1-weighted (TR/TE 386/13 ms) sequences and transverse FLAIR (TR/TE/TI 9110/122/2500 ms) and gradient echo (TR/TE 1000/28 ms) sequences. The spinal imaging protocol included sagittal and dorsal T2-weighted (TR/TE 2880/111 ms) and T1-weighted (TR/TE 623/1 ms), transverse T2-weighted (TR/TE 3290/99 ms) and T1-weighted (TR/TE 651/12 ms) and sagittal STIR (TR/TE/TI 3310/61/140 ms) sequences. The sagittal and dorsal spinal sequences were performed from C1 to T3 (vertebral body), and the transverse sequences used C1 to C7 (vertebral body). Gadolinium (0.1 mmol/kg; 0.045 mmol/lb) was administered intravenously, and post-contrast transverse T1-weighted sequences of the brain and dorsal and sagittal T1-weighted sequences of the spine were acquired. Descriptions of intensity referred to normal appearance of grey matter. The spinal MRI studies showed bilateral symmetrical hyperintensities in the region of both lateral funiculi on transverse T2-weighted images (Figure 1). The lesions were most visible on the transverse sections; they appeared well demarcated and ovoid and extended from the level of the first to the sixth cervical vertebral body (Figure 2). In T1-weighted plain images, the lesions were isointense, and no contrast enhancement was observed. MRI studies of the brain failed to reveal any abnormalities.

Routine CSF analysis (cisterna cerebellomedullaris) with leukocyte (0/μl; reference range 0- 5/μl) and erythrocyte counts (4/μl), CSF cytology and protein measurements (0.18 g/l; reference range 0–0.3 g/l) were unremarkable, as were the lactate concentrations in the CSF (1.6 mmol/l; reference range 0.2-3.1 mmol/l [21,22]) and serum (1.0 mmol/l; reference range 1.1-3.3 mmol/l [22]). No abnormal spontaneous activity pattern was observed during electromyographic recordings using a concentric needle electrode in the anesthetised dog. The tibial and ulnar motor nerve conduction velocity, tibial nerve F-waves and repetitive nerve stimulation were within established laboratory reference ranges.

Considering the progressive clinical course and the MRI lesion pattern, a neurodegenerative disorder predominantly involving the cervical spinal cord white matter with a bilateral and symmetrical distribution was suspected. Due to the existing severe neurological signs, the progressive deterioration and the poor prognosis, the dog was euthanised.

**Figure 3:**
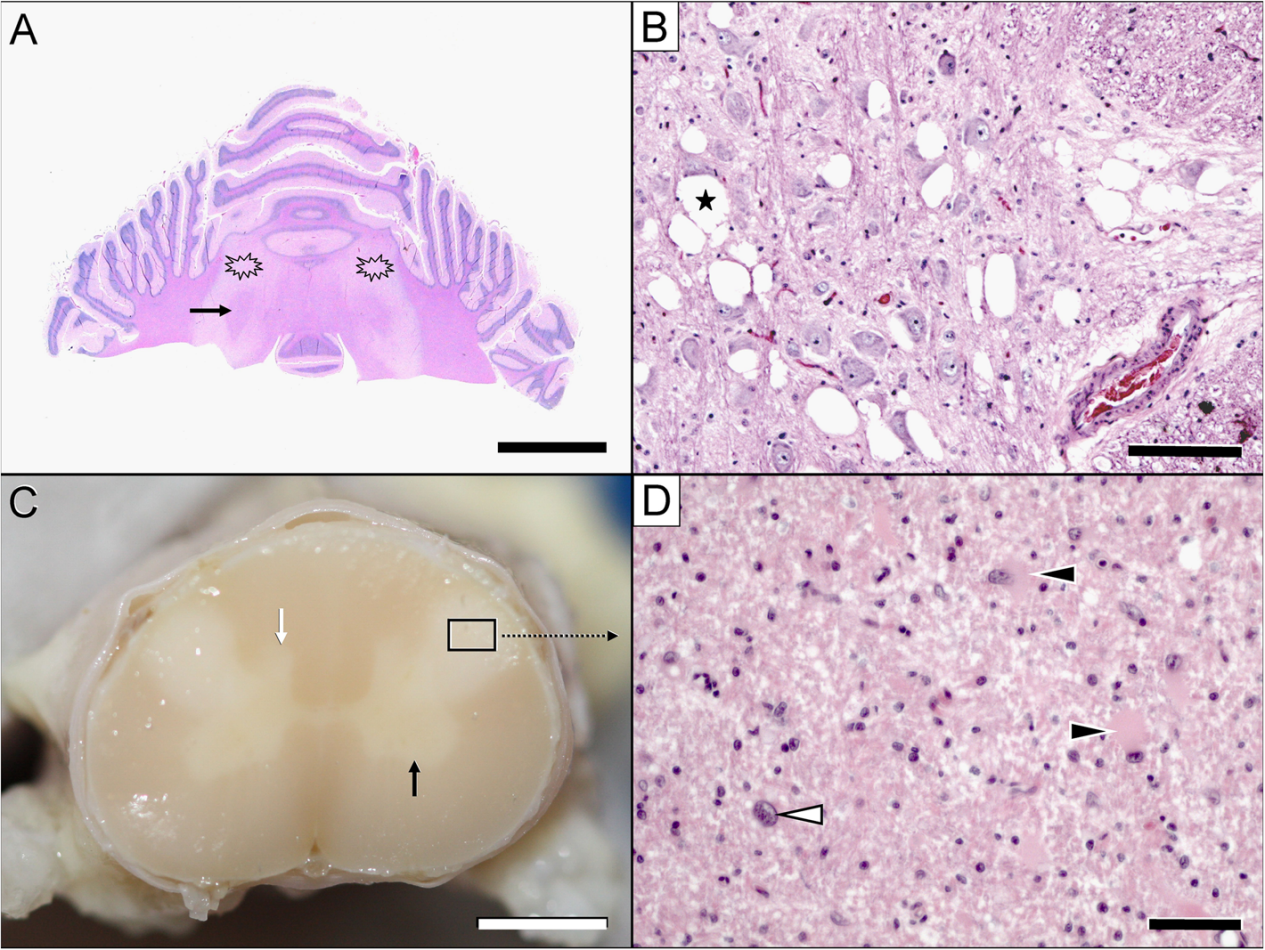
**Pathological lesions in the brain (A, B) & spinal cord (C, D).** The most severe white matter lesions were observed in the cerebellum (**A**: asterisk) and cervical spinal cord (**C**: framed area). Macroscopic examination revealed bilateral, symmetrical lesions in the lateral funiculi of the cervical cord segments only. In transverse sections, these lesions appeared as well-demarcated, whitish, opaque discoloured areas (**C**: framed area). The cerebellar lesions spared the fibres adjacent to the roof nuclei (**A**: arrow). Nuclear degeneration was most severe in the raphe nuclei (**B**) and medial vestibular nuclei (not shown). Note the extensive juxtaneuronal vacuolisation (**B**: asterisk). The affected spinal cord segments show demyelination, astrogliosis and astrocytosis (**D**: white arrowhead) with gemistocytes (**D**: black arrowheads). Within the grey matter, hypoplasia of the dorsal and ventral horn (**C**: black arrow) is evident. Scale bars: A: 1.5 cm; B: 100 μm; C: 2 mm; D: 35 μm.

A complete necropsy was performed, and it confirmed Rottweiler LEM. Significant lesions were confined to the central nervous system. Macroscopic examination revealed bilateral, symmetrical lesions restricted to the dorsal aspect of the lateral funiculi of the cervical spinal cord segments. In transverse sections, these lesions appeared as well-demarcated, whitish, opaque discoloured areas (Figure 3). No gross changes were observed in the brain. Formalin-fixed and paraffin-embedded tissue samples of the brain and spinal cord were sectioned at 5 μm and stained using haematoxylin-eosin and Luxol Fast Blue for histological examination. Histologically, the cervical spinal cord (from C2 to C6 (vertebral body)) exhibited severe, bilaterally symmetrical funicular disruption of the inner dorsal part of the lateral funiculus, including the rubrospinal tract, the innermost layer of the dorsal spinocerebellar tract and the dorsal aspects of the lateral fasciculus proprius. Upon low-power inspection, the lesion was characterised by a severe loss of myelin staining; at high-power, the lesion resembled a dense core of non-myelinated white matter with extensive astrocytosis and astrogliosis with the occasional observation of bizarre cells surrounded by a rim of spongiotic white matter with fibre degeneration, resorptive lesions, vascular prominence and mild-to-marked angiocentric lymphohistiocytic infiltration. The adjacent cervical grey matter appeared hypoplastic in both the ventral and dorsal horns, but there were no further histomorphological changes. Another severe white matter lesion identified in the cerebellar roof showed focal, bilaterally symmetric tissue necrosis, macrospongiosis due to interlamellar myelin sheath oedema (ballooning) and severe intralesional astrogliosis and astrocytosis accompanied by fibrillary astrogliosis and gemistocytes at the margins. A moderate vascular prominence with endothelial hyperplasia was again observed both in the intra- and perilesional areas. Necrotic areas exhibited macrophage-mediated resorption (Figure 3).

Similar demyelinating lesions were observed in the pyramids and caudal cerebellar peduncles and – to a lesser extent – in the medial lemniscus, optic tracts, crura cerebri and subcortical white matter. Lesions in the central visual pathways projected to the optic nerves and manifested as the degeneration of multiple fibres. Further brain stem changes included macrovacuolar degeneration of the raphe nuclei and medial vestibular nuclei associated with mild gliosis and axonal spheroids. Immunohistochemical staining for canine distemper virus was negative. A mild diffuse endoneurial hypercellularity was observed in the preganglionic aspects of the dorsal roots of the cervical spine. Both the radial and common peroneal nerve presented with a mild dropout of myelinated fibres, as denoted by enlarged subperineurial spaces with myxoid replacement oedema, reduced endoneurial area and decreased myelinated nerve fibre density that was associated with a mild expansion of the endoneurial collagenous matrix. Residual large A (alpha)-type myelinated fibres showed myelin ovoids, consistent with stage II – III Wallerian degeneration, and abundant internodal and paranodal inner and outer myelin loops due to the moderate axonal atrophy of the respective fibres.

Due to the phenotypic similarities between human LBSL patients and LEM-affected Rottweilers, the *DARS2* gene was investigated as a candidate for canine LEM. Genomic DNA was extracted from blood collected in tubes containing EDTA using DNeasy blood spin columns (Qiagen). For the *DARS2* mutation analysis, suitable PCR products were amplified using AmpliTaq Gold 360 (Life Technologies). The PCR products were resequenced after rAPid alkaline phosphatase (Roche) and exonuclease I (New England Biolabs) treatment using both PCR primers and the ABI BigDye Terminator Sequencing Kit 3.1 (Life Technologies) in an ABI 3730 sequencer (see Additional file 2: Table S1). The sequence data were analysed using Sequencer 4.9 software (GeneCodes). The sequences of all 17 coding exons and flanking intron sequences of the *DARS2* gene from the affected Rottweiler were identical to a canine reference genome sequence (CanFam3 assembly; http://genome.ucsc.edu).

## Conclusion

Magnetic resonance imaging has become the primary tool for the ante mortem diagnosis of white matter disease in humans due to its high sensitivity for detecting changes in white matter. Decreased myelin and elevated water content is revealed by increased T1 and T2 relaxation times, with a consequent reduction in signal intensity in T1-weighted images and increased signal intensity in T2-weighted images [23]. Thus, the pattern of MRI changes is very helpful in defining disease because it reveals the distribution of histopathologic changes [24,25].

At present, there are few case reports describing the use of MRI for the diagnosis of canine and feline neurodegenerative diseases. T2-weighted hyperintensities of white brain matter were evident in cats with GM2 gangliosidosis [26,27] and in a West Highland white terrier with globoid cell leukodystrophy [28]. Increased signal was also evident in T2-weighted images of the spinal cord of Leonberger dogs with leukoencephalomyelopathy [29]. Dogs with GM2 gangliosidosis displayed T2-weighted hyperintensities in the region of the caudate nucleus and atrophy of the cerebrum and cerebellum [30,31]. MRI of Papillon dogs with neuroaxonal dystrophy [32] and Scottish Terriers with hereditary cerebellar degeneration demonstrated atrophy only and failed to detect changes in white matter [33].

MRI of the cervical spine may be used to support the clinical diagnosis of LEM in Rottweilers. A similar MRI pattern has been described in Leonberger dogs with LEM [29]. Interestingly, however, brain lesions were not detected using MRI in the Leonberger dogs or in the case reported here, although histological analyses showed that the optic tracts and particularly the cerebellar medulla were significantly affected in both breeds [2,29]. It is possible that the white matter lesions in the brain were less advanced than those in the spinal cord at the time of imaging, and improved imaging protocols may be required for the visualisation of brain lesions. These protocols may include smaller slice thicknesses and the application of sequences other than conventional T1- and T2-weighted imaging, e.g., diffusion tensor imaging, magnetisation transfer imaging or MR spectroscopy [34]. It is also questionable whether the pathological changes in these regions have sufficiently altered the physics of the tissue to induce changes visible with a 1.5 T clinical scanner.

Many inherited white matter diseases and associated genetic defects have been described in humans [35]. Leukoencephalopathies may be characterised as lysosomal or peroxisomal disorders, mitochondrial disorders, methylation cycle disorders, organic acidaemias or amino acid disorders or as leukoencephalopathy associated with calcification, hypomyelination, abnormal lipid metabolism, vasculopathy or muscular dystrophy. Many distinct entities, e.g., Alexander’s disease, adult onset autosomal dominant leukoencephalopathy, vanishing white matter disease and adult polyglucosan encephalopathy, have also been recognised. A vast number of genetic defects are currently associated with these conditions, and many more remain to be elucidated; the molecular cause remains unknown in ~50% of affected humans [35]. The lesion distribution and MRI appearance of LBSL are considered unique and diagnostic in humans; consequently, only a single candidate gene was examined in the present study [36].

Leukoencephalopathy with brain stem and spinal cord involvement is a rare, autosomal recessive disorder that typically manifests in childhood or adolescence. The diagnosis of LBSL in humans is based on clinical presentation and is characterised by a slowly progressive cerebellar ataxia, spasticity, dorsal column dysfunction and a highly characteristic pattern of abnormalities observed using MRI and spectroscopy. Typical MRI findings include a combination of high T2-weighted signal changes in the cerebral white matter accompanied by the selective involvement of the brain stem and spinal cord tracts (the entire length of the pyramidal tracts with the additional involvement of cerebellar connections and the intraparenchymal and mesencephalic parts of the trigeminal nerve) [13,37]. MR spectroscopy demonstrates an elevation in lactate levels in the abnormal white matter of almost all of the affected human patients. These findings led researchers to assume that the disease was a mitochondrial disorder, which was subsequently confirmed by the discovery of various mutations in the *DARS2* gene, which encodes mitochondrial aspartyl-tRNA synthetase [14,38]. As demonstrated by multiple case reports of LBSL in humans, normal CSF and blood lactate concentrations, as were noted in the case reported herein, do not exclude a mitochondrial disorder as the underlying cause of leukoencephalomyelopathy. Thus, further investigations should utilise MR spectroscopy to investigate the possible mitochondrial origin of Rottweiler LEM.

The diagnosis of mitochondrial disorders faces specific difficulties due to the complex genetics of these conditions. Mitochondrial disorders may occur due to mutations in mitochondrial genes or mutations in nuclear proteins, with mitochondrial tRNA representing a hot spot for mutations. Heteroplasmy, i.e., the simultaneous presence of mutated and normal RNA/DNA in the cell, is a characteristic feature of mitochondrial disorders. The degree of heteroplasmy varies between tissues in the same organism, which is considered a critical factor in the manifestation of mitochondrial disease in specific tissues [36,39]. Finally, we investigated the coding region of the canine *DARS2* gene as a candidate causative gene for LEM, and no mutation was found. At this time, we cannot rule out the possibility of variants in the promoter or intronic regions that could affect *DARS2* expression. More comprehensive DNA sequencing approaches, such as the use of next-generation technologies for whole-exome or whole-genome resequencing, may enable the identification of the causative mutation of Rottweiler LEM. A recent study identified the causative mutation of canine neonatal cerebellar cortical degeneration in SPTBN2 (genome-wide mRNA sequencing) using only a single case of this neurodegenerative disease [40].

Further limitations of our case report include the lack of pedigree analysis and brain lactate MR spectroscopy measurements and the failure of MR to demonstrate the involvement of the cerebrum despite the pathology observed in histological sections. Another limitation is that the comparison of the pathologies of these diseases in dogs and humans is limited by the paucity of case data from both. To date, there is only one short description of the pathology of LBSL in humans which has shown spongy white matter degeneration, rarefaction of the neuropil, macrophage infiltration and an increased number of astrocytes in the white matter of the brain and axonal degeneration of the peripheral nerves [25]. Spinal cord changes have not been noted, but it is unknown whether this part of the CNS was sampled and investigated. In dogs with LBSL-like changes, the neuroanatomical mapping of CNS lesions is more precise [2,29]. Clinical and pathological findings emphasise cerebellar and spinal changes, although the microscopic white matter damage is far more widespread and extends from the lower brain stem to the subcortical white matter. Consistent with the fibres affected, Gamble et al. discovered secondary grey matter changes in connected brain stem nuclei, such as the accessory cuneate nucleus, nucleus gracilis, nucleus cuneatus and nucleus of the dorsal spinocerebellar tract [1]. However, the involvement of multiple independent centres and tracts is compatible with multisystemic degeneration, as has been shown in Leonbergers and Rottweilers (discussed above) [2,29]. We also discovered macrovacuolar nuclear degeneration in the Rottweiler, which has not previously been described in dogs. Vacuole formation in LBSL patients was predominantly perineuronal and was therefore dissimilar to the neuronal vacuolation and spinocerebellar degeneration observed in Rottweiler dogs [16]. This degeneration merits further examination to investigate the relationship between neurons and astrocytes in the subcortical grey matter. It also remains to be established whether this manifestation causes the white matter pathology or whether it is an additional, co-existing disorder that is distinct from the breed-specific neurodegenerative disorders described above.

In summary, magnetic resonance imaging revealed leukodystrophic lesions in the lateral funiculi of the cervical spinal cord; these findings will assist in the ante mortem diagnosis of future cases of suspected LEM in Rottweilers. Further investigations should utilise MR spectroscopy to investigate the possible mitochondrial origin of Rottweiler LEM. Although LEM is similar to LBSL based on its clinical features and imaging results, we were unable to identify a coding or splice site mutation in the canine *DARS2* gene in our case, suggesting that other genes may be involved in Rottweiler LEM and potentially also in human LBSL.

## Abbreviations

CSF: Cerebrospinal Fluid; FLAIR: Fluid-Attenuated Inversion Recovery; LBSL: Leukoencephalopathy with Brain Stem and Spinal Cord Involvement; LEM: Leukoencephalomyelopathy; MR: Magnetic Resonance; MRI: Magnetic Resonance Imaging; Ms: Millisecond; TE: Time to Echo; TR: Time to Repetition.

## Competing interests

The authors declare that they have no competing interests.

## Authors’ contributions

KH was responsible for data collection and interpretation and for drafting the manuscript. KM performed the necropsy and histopathology and provided histopathology images. KF performed all diagnostic imaging procedures and selected the appropriate images. MD and CD conducted the genetic study. BR contributed substantially to the acquisition of data used in the manuscript. AF contributed to data collection, helped draft the manuscript and finalised the version to be published. All authors have approved the final manuscript.

## Supplementary Material

Additional file 1 **Movie of a Rottweiler with confirmed leukoencephalomyelopathy.** The movie shows the severe generalised ataxia with hypermetria of the thoracic (with prolonged protraction, overreaching action and limb extension) and pelvic limbs. The postural reactions were delayed, and the thoracic limbs were more severely affected than the pelvic limbs. Click here for file

Additional file 2 Sequencing methods and primers. Click here for file
