# Supplementary material for: Magnetic resonance imaging and genetic investigation of a case of rottweiler leukoencephalomyelopathy
Source: BMC Vet Res. 2013 Mar 26;9:57. doi: 10.1186/1746-6148-9-57 (PMC3614464; doi:10.1186/1746-6148-9-57)
Supplement: Additional file 2 — Sequencing methods and primers. [file 1746-6148-9-57-S2.pdf]

DARS2	primer	sequence	product size (bp)
exon 1	F1	AAGGCCCCAGATTACTTGTAGA	519
	R1	AAGGCAGAATTCATGTGGTT	
exon 2	F2	ACAGTAAGACTGTTAATTCACTTGGTT	490
	R2	TCATTTATTTATTCATGAGAGACACAG	
exon 3	F3	AAAAGTAAATGCAAAGTCTTTTTGT	456
	R3	TTGTCTGTGATATGAGTTGCACA	
exon 4	F4	TTTAACTTGCTGTTTCATGTGGA	480
	R4	AACATGCCTACTCACATTGGTG	
exon 5	F5	TGAGCTCATCAGCCACAGAT	480
	R5	AACCTGTCTGATAAAGCAGCCTA	
exon 6	F6	CAAACTTAGAAGTTGTAGGAATTAAGG	480
	R6	TTAACCTTTCTTCACTGCTTTACA	
exon 7	F7	TGACAGAAGTATCACCTTATGACCA	437
	R7	AAAGCAGACGCTCAACCACT	
exon 8	F8	GAATTGGGAATTCATTTCAACA	610
	R8	AAGGCAGCGCTAAACCACT	
exon 9	F9	AATTTCTTATGTCCTTTAGGTACCATT	808
	R9	GATCCCTGGGTGGCACAG	
exon 10	F10	CAAACCCCCACCCATGTAT	480
	R10	GTACTGTAGTGCTGACATGCTATTATT	
exon 11	F11	GCCCATGGTAAACACTGTGA	499
	R11	AGGGTCTGATCCTGGAGACC	
exon 12	F12	CATTCATAGTTGTTTCCAGTGAGG	500
	R12	AGGATCGAATCCCATGTCAG	
exon 13	F13	TTTTTATTCCCTTCTGTTTTTGA	521
	R13	TGATACAATGCATTATTTAGTGCTCA	
exon 14	F14	TGCCCAGTCACTGTTACCAA	608
	R14	TTTCTGTTCTACAGGTTGAAGTCAC	
exon 15	F15	AGGAAATGTGGCAACAAAGC	506
	R15	CGGTCATTTACTTTTCAGTCTTACA	
exon 16	F16	CCTGGCTACATTTACTGAGTGC	459
	R16	TGCATTTTAAACCTGTATCAACG	
exon 17	F17	GAAAAGAACCTTTCAGACAGACAGA	562
	R17	TCCCCAAAAATCCTAATAATGC	
exon 18	F18	AACTGCATTACATGCCAGTCTA	429
	R18	AAATTTACTGCCCAGTCTTGAAA	
